# Relationship of Vessel Density to Vessel Length Density in Patients with Treated Fabry Disease

**DOI:** 10.3390/diagnostics13071227

**Published:** 2023-03-24

**Authors:** Maximilian Robert Justus Wiest, Mario Damiano Toro, Albina Nowak, Anahita Bajka, Katrin Fasler, Mayss Al-Sheikh, Timothy Hamann, Sandrine Anne Zweifel

**Affiliations:** 1 Department of Ophthalmology, University Hospital Zurich, University of Zurich, 8091 Zurich, Switzerland; 2 Eye Clinic, Public Health Department, Federico II University, 80131 Naples, Italy; 3 Chair and Department of Ophthalmology with Pediatric Service, Medical University of Lublin, 20079 Lublin, Poland; 4 Department of Endocrinology and Clinical Nutrition, University Hospital Zurich, University of Zurich, 8091 Zurich, Switzerland; 5 Department of Internal Medicine, Psychiatry University Clinic Zurich, 8091 Zurich, Switzerland

**Keywords:** Fabry disease, optical coherence tomography angiography (OCTA), OCTA parameters, lysosomal storage disorder, vessel density, vessel length density

## Abstract

Background: Fabry disease (FD) is a potentially lethal lysosomal disorder with systemic vascular changes. Previous studies demonstrated retinal vascular involvement using optical coherence tomography angiography (OCTA) in affected patients; Aim: To analyze and quantify the retinal vasculature measuring vessel density (VD), vessel length density (VLD), and the ratio of VD to VLD (VD/VLD) in superficial capillary plexuses (SCP) and deep capillary plexuses (DCP) using OCTA in patients with FD and to show whether they differ from healthy controls (HC); Patients and methods: Single-center, retrospective, consecutive cohort study of patients with genetically proven FD. Patients underwent an ophthalmological examination including OCTA. VD, VLD, foveal avascular zone (FAZ), and the VD/VLD were compared to an HC group using a linear mixed model; Results: A statistically significant difference in the VLD and VD/VLD of DCP was observed between the two groups (*p* < 0.001). Using ROC curves with AUC and Youden’s Index, a cut-off value for differentiating both groups using VD/VLD in DCP FD with high specificity and high sensitivity was established; Conclusions: FD and HC groups seem to be separable using the VD/VLD ratio in DCP. This new biomarker might differentiate changes in the retinal microvasculature that are not detectable by VD or VLD alone.

## 1. Introduction

Fabry disease (FD) is a lysosomal storage disorder in which a decrease in the overall activity of the enzyme α-galactosidase (α-Gal A), due to mutations in the responsible α-galactosidase gene [1], leads to the accumulation of globotriaosylsphingosine (Gb3) in fluids and lysosomes of affected patients [2]. This accumulation of Gb3 in organs and tissues can cause progressive organ or tissue dysfunction [3]. The most common complications of FD are cardiovascular disease, premature stroke, and renal failure as well as frequent pain crises [1,2,3,4,5].

Fabry disease is heritable in an X-linked recessive fashion due to genetic variants in the GLA gene [6,7]. In male patients, two distinct subclasses of the disease have been identified: the early-onset, classic phenotype, characterized by low α-Gal A activity, can be differentiated clinically from the later-onset phenotype with residual α-Gal A activity by early symptoms, such as acroparesthesias, abdominal cramping, pain crises, and hypohydrosis. The later-onset phenotype typically presents with hypertrophic cardiomyopathy or chronic kidney disease later in life, during adulthood [1,8,9]. Phenotypes in females are more heterogenous due to random x-chromosomal inactivation, with high variability in residual α-Gal A activity, thus presenting with symptoms ranging from very severe to very mild [10].

Ophthalmic manifestations are among the most common early manifestations of FD and can be key in early diagnosis [11]. The most commonly known ocular finding is cornea verticillata (CV), an often bilateral, vortex-like opacity found in the cornea [12,13,14]. A spoke-like posterior cataract is another specific finding which can be observed in about 21.9% of males and 9.8% of females with FD. This spoke-like posterior cataract is one of the few pathognomonic clinical findings that can be observed noninvasively [15,16].

Recently, intravenous enzyme replacement therapy (ERT) has become available for patients suffering from FD [17,18,19]. It has been reported that LysoGb3 levels, as well as symptoms, decrease under ERT [20,21].

Optical coherence tomography angiography (OCTA) is a non-invasive, non-contact, infrared laser-based imaging technique that has become increasingly available for clinical and scientific use in the past years [22]. Through it, highly detailed functional and structural examinations of the retinal vasculature are possible in a non-invasive manner [23]. OCTA can generate quantitative information such as the vessel density (VD) and vessel length density (VLD) of a given retinal volume [24,25]. These are among the most common parameters used for the detection of differences in the retinal vasculature using OCTA, although it has been recognized by some authors, that these parameters only provide partial information about the actual state of the retinal vasculature [26]. Previous studies have addressed this issue, but to date, there is no comprehensive single OCTA parameter replacing VD and VLD, which are still the most commonly used parameters in the scientific literature [26,27,28,29,30,31,32,33].

Previous publications have demonstrated differences in VD and VLD between patients suffering from FD and healthy controls in OCTA [30,34,35,36]. Furthermore, it has been shown, that OCTA parameters can be associated with systemic biomarkers of FD, for example, echocardiographic parameters in FD patients with myocardial disease [37] and LysoGb3 levels [30]. Thus, OCTA parameters in FD might be useful as surrogate biomarkers for systemic involvement and progression in FD. These findings further highlight the potential use of OCTA as a diagnostic or screening tool in patients suspected of suffering from FD, or other systemic diseases in general [28,38].

This study’s purpose was to analyze and quantify the retinal vasculature by measuring the VD and VLD in en-face images of the superficial capillary plexuses (SCP) and deep capillary plexuses (DCP) using swept-source OCTA (SS OCTA) in patients with FD and to show whether they differ from age-matched healthy controls (HC). In addition to analyzing classical OCTA parameters such as VD and VLD, we propose a new OCTA biomarker, VD/VLD, which is calculated from the ratio of VD to VLD, as a more comprehensive tool for evaluating retinal abnormalities in patients suffering from systemic diseases using OCTA.

## 2. Materials and Methods

This is a retrospective, monocentric analysis of a cohort of consecutive, genetically proven FD patients who undergo annual ophthalmological checkups at the Department of Ophthalmology, University Hospital of Zurich, Switzerland. The recruitment period was from 1 December 2017 to 9 September 2020. The responsible institutional review board gave approval for this study (Kantonale Ethikkommission, Canton of Zurich, BASEC-Nr. 2019-02043). Informed consent for the use of personal data was acquired in written form from each patient. The tenets of the Declaration of Helsinki were adhered to.

The following criteria were used for inclusion: minimally 16 years of age and a genetically confirmed FD diagnosis. The criteria of exclusion were: FD-unrelated systemic or ocular disease, previous or current retinal or macular vascular disease, history of glaucoma, significant opacity of the lens, a myopia of more than −4 diopters, and insufficient (signal strength of 8/10 or lower) quality of OCTA scans. As controls (healthy group), age-matched healthy individuals without a systemic vascular or ocular disease who otherwise fulfilled all the above inclusion or exclusion criteria were included.

All patients underwent an ophthalmological examination with best corrected visual acuity (BCVA), intraocular pressure (IOP) measurement, slit lamp examination of the anterior segment and biomicroscopy of the fundus, ultrawide-field scanning laser ophthalmoscopy (Optomap, Optos, Marlborough, MA, USA), spectral-domain optical coherence tomography (SD-OCT) and swept-source OCTA.

For BCVA, an auto-refractometer (NT-530/510^®^, NIDEK Inc., San Jose, CA, USA) was used for refraction. BCVA was measured using the acquired refractive values and then converted to Early Treatment Diabetic Retinopathy Study (ETDRS) number values [35]. The auto-refractometer also measured the IOP using pneumatic tonometry.

OCTA images were acquired using the swept-source PLEX Elite 9000 device (Carl Zeiss Meditec Inc., Dublin, CA, USA), using the software version 2.0.1.47652. 3 mm × 3 mm image scans with centration on the fovea were acquired by a certified and trained ophthalmologist. Only images with a signal strength of 8/10 or more were included, as it has been shown that signal strength influences quantitative measurements in OCTA [39]. Moreover, scans with bad centration, motion artifacts, incorrect focus, or errors in projection artifact removal were excluded for further analysis. Sample images for HC and FD patients can be appreciated in Supplement Figure S1.

The instrument software supplied by the manufacturer (Macular Density v.0.7.1, ARI Network Hub, Carl Zeiss Meditec Inc., Dublin, CA, USA) automatically generated quantitative parameters of the 3 × 3 mm cube scans using layer segmentation and experimental analysis vascular density quantification software. The generated values are the aforementioned vascular density metrics, VD, representing the fraction of a region with perfusion signal, and VLD, representing the total length of perfused vasculature in a given area in millimeters of perfused vasculature per square millimeter of area, or inverse millimeters (mm^−1^) [40,41]. The innermost rings of the ETDRS grid centered on the fovea with an inner diameter of 1 mm and an outer diameter of 3 mm (inner ring, iR) were analyzed [42].

Age at examination date, FD phenotype, gender, IOP, BCVA, as well as the absence or presence of ophthalmological findings associated with FD, and potential systemic involvement, were acquired by analyzing the electronic health reports and used for statistical analysis.

### Statistical Analysis

SPSS software (version 26, IBM Corporation, Armonk, NY, USA) was used for statistical analysis. OCTA data and Clinical parameters were displayed using descriptive statistics. To test the influence of FD on OCTA parameters, a linear mixed model was applied. This allowed the inclusion of both eyes from each subject, whenever both were available, by correcting for multiple measurements (left eye, right eye, the repeated covariance setting was compound symmetry).

An analysis of the differences in OCTA parameters of both groups was performed. The influence of sex, age, and laterality was accounted for by introducing sex and laterality as cofactors and age as covariance. A *p*-value of less than 0.05 in the type III fixed effect test (F-Test) lead to a detailed review of the estimates of the fixed effect of healthy FD status on the analyzed OCTA-Parameters. This analysis was performed for VLD in the SCP, VD in the DCP, and VD in the SCP and VLD in the DCP.

The statistical analysis was repeated for the following subgroups: female patients and male patients.

## 3. Results

Of the 63 patients identified with FD, 6 were excluded because OCTA imaging of good quality was unavailable. Of the remaining 57 patients with 114 eyes, 5 eyes were excluded because of bad focus or incorrect centration (2 and 3, respectively). Amongst the remaining 109 eyes, one DCP segmentation was excluded due to a failure in segmentation. Eighty-four patients (46 female, 38 male) with 117 (59 left, 58 right) eyes were included in the healthy group.

For the final analysis, 57 (34 female, 23 male) patients with genetically proven FD with 109 (52 left, 57 right) eyes and 108 DCP and 109 SCP segmentations were included. Forty-one patients with the classic phenotype (14 male, 27 female) and 16 suffering from the later-onset phenotype (9 male, 7 female) were included. Forty patients (18 female, 22 male, 70.2% overall) were under ERT. The mean age was 43.4 (± 15.3) years in FD and 38.6 (± 14.7) years in the healthy group.

Cornea verticillata and retinal vessel tortuosity were observed in 74 (67.9%) and 52 (47.7%) of FD eyes, respectively. No FD eyes with a spoke-like posterior cataract were observed. Mean BCVA and IOP were 85.32(± 4.92) ETDRS letters and 14.8(± 2.9) mmHg, 86.75(± 3.37) ETDRS letters and 15.1(± 1.70) mmHg in FD and healthy group respectively. A summary of demographics, genetic, and clinical characteristics are shown in Table 1 and Table 2.

The mean VD of the SCP was 0.38 ± 0.02 and 0.38 ± 0.03 in the FD group and healthy group respectively. For the DCP, the mean VD was 0.28 ± 0.05 and 0.29 ± 0.05 respectively. The mean VLD was 17.2 mm^−1^ ± 1.16 mm^−1^ and 17.5 mm^−1^ ± 1.20 mm^−1^ in the SCP and 11.0 mm^−1^ ± 1.79 mm^−1^ and 13.5 mm^−1^ ± 2.03 mm^−1^ in the FD group and healthy group respectively. Statistically significant differences were detected in VLD for DCP (*p* < 0.001). No statistically significant results for VD in SCP and DCP and VLD in SCP between the FD group and the healthy group were detected. A statistically significant difference could also be detected in the VD/VLD for the DCP, with a mean value of 45.7 ± 2.39 in the FD group and 47.2 ± 1.36 in the healthy group (*p* < 0.001).

These results are summarized in Table 3.

**Figure 1:**
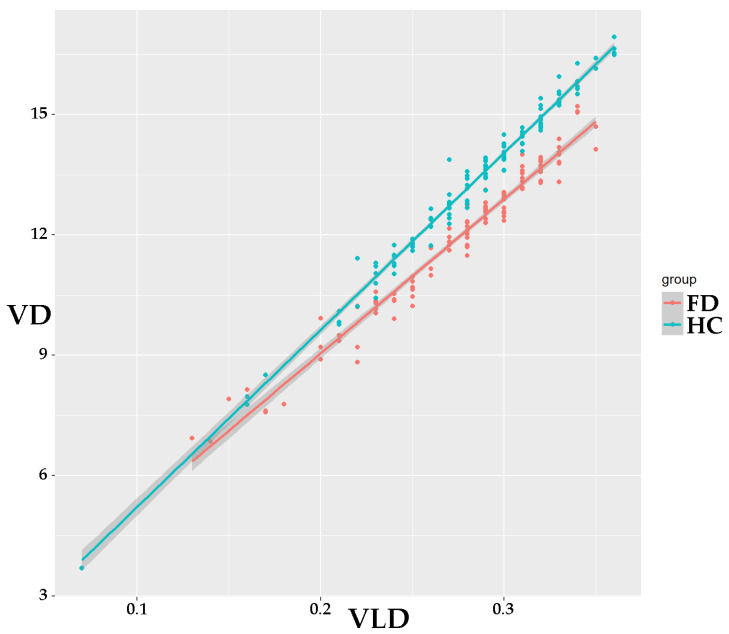
Scatterplot for VD/VLD in DCP ratio in FD and healthy cohort. A near-linear relationship separating FD from the healthy cohort can be observed, suggesting the use of VD/VLD in the DCP ratio for a more distinct separation between study groups. Abbreviations: VD: vessel density; VLD: vessel length density; DCP: deep capillary plexus; FD: Fabry Disease; HC: healthy cohort.

When visualizing VD to VLD ratio in SCP and DCP, a highly linear relationship between disease status (FD or healthy group) was observed in the VD/VLD ratio for the DCP (Figure 1). No such effect was observable in the VD/VLD ratio for SCP.

**Figure 2:**
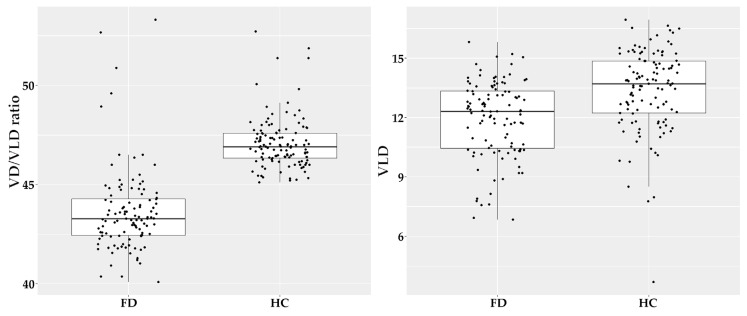
Boxplots for VD/VLD in DCP ratio and VLD in DCP in FD and healthy cohort. Abbreviations: VD: vessel density; VLD: vessel length density; DCP: deep capillary plexus; FD: Fabry Disease; HC: healthy cohort.

The ratio between VD and VLD was calculated and visualized in boxplots, which are shown below (Figure 2).

**Figure 3:**
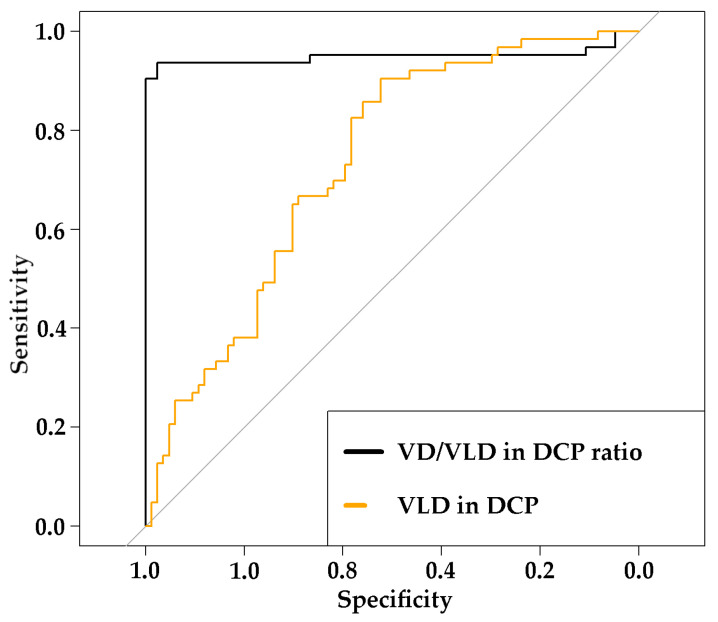
ROC curve for VD/VLD in DCP ratio and VLD in DCP in the detection of FD. Abbreviations: VD: vessel density; VLD: vessel length density; DCP: deep capillary plexus.

In order to explore the discriminating ability of VD/VLD in DCP, receiver operating characteristic (ROC) curves with corresponding area under the curve (AUC) and 95%-confidence intervals (CI) were calculated and compared to ROC curves and AUCs with 95%-CI’s of VLD in DCP, which was the only single OCTA parameter where a significant difference in between FD and the healthy group was observed (Figure 3).

Using Youden’s Index for rating diagnostic tests [43], the best cut-off with specificity and sensitivity for detecting FD was calculated for VD/VLD in DCP and VLD in DCP, respectively. For VD/VLD in DCP, the best cut-off was 45.51, with a high specificity of 0.976 and a high sensitivity of 0.937. For VLD in DCP, the best cut-off was 13.82 mm^−1^ with a specificity of 0.523 and a sensitivity of 0.905, indicating moderate performance at identifying FD.

## 4. Discussion

In this study, we observed a statistically significant difference in the VLD of DCP between the FD and HC groups. In our mixed-effect models, we were able to clearly separate the FD and HC group based on the VD/VLD ratio in DCP. Using ROC curves with corresponding AUC and Youden’s Index we were able to show that VD/VLD could differentiate FD from HC patients with high specificity and high sensitivity at a cut-off value of 45.51 in our study population.

Previous publications demonstrated that OCTA is able to detect alterations in the retinal microvasculature and could potentially be useful as an early diagnostic biomarker and for the follow-up examinations of patients affected by different systemic diseases [28,30,38,40,44,45]. In general, VD and VLD are OCTA biomarkers that have been used to assess the retinal microvasculature in different retinal and systemic diseases for years [24,28,29,46,47,48,49,50,51,52]. While numerous findings on differences in VD and/or VLD in multiple diseases have been reported this study is the first to report on a statistically significant difference between an affected population (our FD cohort) and an HC group in VD/VLD ratio in DCP. This is the first publication describing VD/VLD as an imaging biomarker in OCTA. This new biomarker might be more sensitive to architectural changes in the retinal microvasculature as it is not only a reflection of the total scan area covered by flow signal (VD) or the total length of detected vasculature by square millimeter (VLD) but of both and might be able to highlight changes in the relationship of the two aforementioned, already well established OCTA parameters. Especially in systemic diseases such as FD, where fundamental changes in the histology of vascular tissues occur through the continuous deposition of Gb3 [2,15], a more comprehensive biomarker, such as the VD/VLD ratio, might be more sensitive than traditional biomarkers. In our cohort, FD patients showed lower VD values at the same VLD values as our HC patients. As VLD represents the total length of observed vasculature with flow signal and VD represents the total area of detected perfusion on OCTA, this might suggest, that healthy controls have a higher coverage of perfused retina per the same total amount of vessel length than FD patients. This could indicate a lower “quality” of microvasculature in FD patients. As this is the first publication to investigate the relationship of VD to VLD in FD patients and HC patients, these findings, and the role of VD/VLD to assess perfusion quality need to be investigated further.

The clinical manifestation of FD varies substantially, depending on the remaining activity of α-Gal A [1]. Clinical findings usually range from the mild, often initially non-system involving later-onset phenotype to the severe, classic phenotype with almost no residual enzyme activity [1]. In a previous publication [30], we have shown, that OCTA parameters are associated with lysoGb3-levels in FD patients. These findings support the growing evidence that OCTA indeed could be used as a diagnostic biomarker in FD patients. Dogan et al. [53] have shown, that VD in DCP is significantly lower in FD patients when compared to a healthy cohort. In our study, we did not find a difference in VD, but in VLD between FD and HC (*p* < 0.001). As Dogan et al., we were not able to show any significant differences in the SCP, as other groups have reported [34]. When examining the VD/VLD ratio in DCP, we detected a statistically significant difference between the FD and HC groups, with the FD group having a lower VD/VLD ratio than the HC group. One possible explanation for the DCP being more affected than the SCP has already been reported and discussed by Dogan et al. [53]. According to these authors, the DCP has been demonstrated to be the more severely affected vascular plexus when compared to the SCP in events of increased hydrostatic pressure after the capillaries such as retinal vein occlusions. The SCP, due to its more direct connections to the retinal arterioles, carries high-pressure fluid, and higher mean intravascular pressure, and is therefore more resistant to increased hydrostatic pressure down the line [54]. In FD, continuous accumulation of Gb3 in the vascular tissue leads to a thickening of vessel walls and, over time, to an increase in vessel tortuosity. In theory, this increase in flow resistance could lead to a decrease in flow in the more susceptible DCP [15]. The authors postulate that these abovementioned factors discussed in light of retinal vein occlusions might translate to FD patients and explain the increased vulnerability of and decreased VD values in the DCP they observed in their FD cohort [53]. While we were unable to detect any statistically significant difference apart from VLD in DCP in our cohort, we were able to clearly separate the FD group from the HC group using the VD/VLD ratio in DCP, which might support the previously discussed theory of increased susceptibility of the DCP to increased flow resistance down the line.

To date, despite the advances in the treatment and diagnosis of FD, scientific evidence of associations between systemic findings and OCTA parameters is scarce and only a few studies investigate whether these associations exist [30,34,53]. Cennamo et al. [34] have shown that the VD of both the DCP and SCP inversely associate with different echocardiographic parameters (Systolic pulmonary arterial pressure, E/e’ ratio, intraventricular septal thickness, and left atrial volume index). Lin et al. [55] reported in a recent publication about 26 patients with FD that cystatine C and serum creatinine are associated inversely with VD. Our previous findings show that lysoGb3 seems to be inversely associated with both VLD and VD in the DCP and SCP [30]. Our study adds to this as of yet still very limited understanding of the relationship between FD, disease severity, and its impact on the retinal vascular structure detectable by OCTA.

This study has major limitations such as its limited sample size, which makes correlations with other parameters than disease status, such as disease severity or systemic features difficult. Other limitations include the retrospective nature of this study and the imbalance of male-to-female patients in an X-linked disease. Furthermore, this study only considered the central retinal. This might not take into account other retinal alterations of the microvasculature which could be detectable in the retinal periphery.

## 5. Conclusions

In conclusion, our study shows that FD patients and HC can be clearly separated using the VD/VLD ratio in the DCP. To our knowledge, this is the first publication to highlight the VD/VLD ratio for differentiating healthy and FD patients. This new biomarker might be able to detect subtle differences in the retinal microvasculature that are not objectifiable by VD or VLD alone. In addition, this study adds further insight into the relationship between VD and VLD in both SCP and DCP.

While the evidence that OCTA might yield useful biomarkers for FD and systemic diseases, in general, is increasing, more data is still necessary to fully understand changes in the retinal vasculature brought by FD.

Hence, further prospective controlled trials with a longer follow-up, able to better assess dynamic changes in OCTA parameters, and a larger sample size are in demand.

## Figures and Tables

**Table 1:** Demographics and clinical characteristics of both study cohorts.

		FD Patients *n* = 57	Healthy Patients *n* = 84
gender	male	23	38
	female	34	46
mean age (years) ± SD		43.4 ± 15.3	38.6 ± 14.7
FD phenotype	classic	41	n/a
	later-onset	16	n/a
enzyme replacement therapy *	yes	40	n/a
	no	17	n/a

*n*: number; * agalsidase beta or ANN-agalsidase alfa.

**Table 2:** Ocular characteristics of the study cohort.

		FD Eyes *n* = 109	Healthy Eyes *n* = 117
laterality	right	57	58
	left	52	59
cornea verticillata	yes	74	0
	no	35	0
retinal vessel tortuosity	yes	52	
	no	57	
mean BCVA (EDTRS letters)		85.32 ± 4.92	86.75 ± 3.37
mean IOP (mmHg)		14.8 ± 2.9	15.1 ± 1.7

*n*: number; BCVA: best corrected visual acuity; ETDRS: Early Diabetic Treatment Retinopathy Study; IOP: intraocular pressure.

**Table 3:** Mean values of OCTA parameters in both study cohorts.

OCTA Parameter		FD Group (Mean + SD)	Healthy Group (Mean + SD)	*p*-Value
SCP	VD	0.38 ± 0.02	0.38 ± 0.03	NS
	VLD	17.2 ± 1.16	17.5 ± 1.20	NS
	VLD/VD	45.8 ± 2.42	46.1 ± 2.45	NS
DCP	VD	0.28 ± 0.05	0.29 ± 0.05	NS
	VLD	12.0 ± 1.79	13.5 ± 2.03	<0.001
	VLD/VD	45.7 ± 2.39	47.2 ± 1.36	<0.001

OCTA: Optical coherence tomography angiography; SD: standard deviation; FD: Fabry Disease; SCP: superficial capillary plexus; VD: vessel density; VLD: vessel length density; DCP: deep capillary plexus, NS: not significant.

## Data Availability

Data will be made available upon request to the corresponding author.

## Supplementary Materials

The following supporting information can be downloaded at: https://www.mdpi.com/article/10.3390/diagnostics13071227/s1, Figure S1: Sample OCTA superficial and deep capillary plexus segmentations of a FD patient and a HC patient.
