# Supplementary material for: Relationship of Vessel Density to Vessel Length Density in Patients with Treated Fabry Disease
Source: Diagnostics (Basel). 2023 Mar 24;13(7):1227. doi: 10.3390/diagnostics13071227 (PMC10093750; doi:10.3390/diagnostics13071227)
Supplement: Supplementary file 1 [file diagnostics-13-01227-s001.zip › diagnostics-2158802-supplementary.pdf]

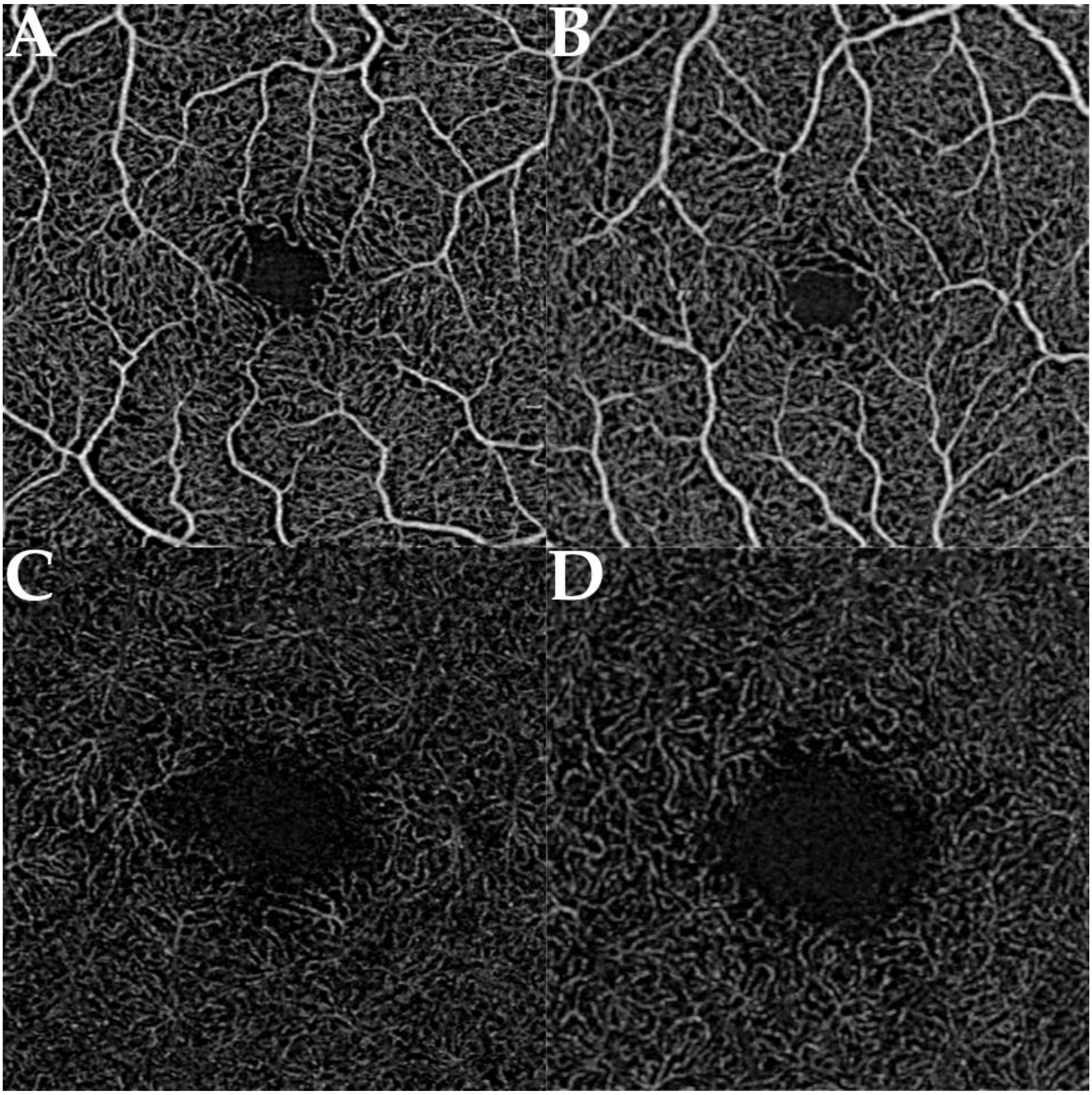

Legend: A: Superficial capillary plexus (SCP) segmentation of a Fabry Disease (FD) patient; B SCP of a healthy control (HC) patient; C: Deep capillary plexus (DCP) segmentation of a FD patient; DCP segmentation of a HC patient.

Supplemental Figure S1. Example Optical Coherence Tomography Angiography Segmentations of a Fabry Disease (FD) and Healthy Control (HC) Patient. In this figure, no macroscopic differences allowing the obvious classification into FD or HC status can be appreciated.
